# Shoulder Arthroplasty Imaging: What’s New

**DOI:** 10.2174/1874325001711011126

**Published:** 2017-09-30

**Authors:** T.M Gregory, J. Gregory, E. Nicolas, J. Pierrart, E. Masmejean

**Affiliations:** 1 Upper Limb Surgery Unit, European Hospital Georges Pompidou, Assistance Publique-Hôpitaux de Paris, University Paris Descartes, Paris, France; 2 Department of Radiology, European Hospital Georges Pompidou, Assistance Publique-Hôpitaux de Paris, University Paris Descartes Paris, France; 3 Department of Mechanical Engineering, Imperial College, London, UK

**Keywords:** Shoulder prosthesis, Radiolucent line, CT-scan, Aseptic loosening, Planning, Patient specific instrument

## Abstract

**Background:**

Shoulder arthroplasty, in its different forms (hemiarthroplasty, total shoulder arthroplasty and reverse total shoulder arthroplasty) has transformed the clinical outcomes of shoulder disorders. Improvement of general clinical outcome is the result of stronger adequacy of the treatment to the diagnosis, enhanced surgical techniques, specific implanted materials, and more accurate follow up. Imaging is an important tool in each step of these processes.

**Method:**

This article is a review article declining recent imaging processes for shoulder arthroplasty.

**Results:**

Shoulder imaging is important for shoulder arthroplasty pre-operative planning but also for post-operative monitoring of the prosthesis and this article has a focus on the validity of plain radiographs for detecting radiolucent line and on new Computed Tomography scan method established to eliminate the prosthesis metallic artefacts that obscure the component fixation visualisation.

**Conclusion:**

Number of shoulder arthroplasties implanted have grown up rapidly for the past decade, leading to an increase in the number of complications. In parallel, new imaging system have been established to monitor these complications, especially component loosening

## 1 INTRODUCTION

The ultimate therapy of primary shoulder osteoarthritis reluctant to medical treatment is total shoulder arthroplasty (TSA). But constant increase in indications (rotator cuff injuries, proximal humerus fracture, bone loss, cuff tear pathologies, revision of already implanted material [1]) and incidence [2] leads to numerous interventions in plan and many patients’ follow-up.

From the diagnosis of the pathology to the long-term follow-up of the implant, imaging is used in many steps of the prosthesis life history.

The goal of this review article is to establish a non-exhaustive list of radiological methods that assist surgeons in the diagnosis of shoulder diseases eligible for shoulder arthroplasty and refine the prediction of clinical outcomes, adapt materials and techniques to design patient specific procedures, and also assist in long-term follow-up of the patients. We separated the processes in two groups: preoperative imaging and postoperative imaging.

## 2 PREOPERATIVE IMAGING

In this review, we will focus on the imaging used in emergency (fractures) and chronic (osteoarthritis, …) shoulder conditions requiring shoulder arthroplasty.

### 2.1 Fracture

Imaging has become a crucial step for the diagnosis and treatment of proximal humerus fractures. However, plain radiographs are not always sufficient, as numerous crucial radio-transparent structures (ligaments, capsule, tendons, muscles) surround the shoulder joint and bone fractures require thorough imaging exams if the radiographs are not conclusive. Thus, Computed Tomography (CT) scan and more rarely Magnetic Resonance Imaging (MRI), when an assessment of soft tissues is required, are used for better understanding of the fracture.

Three-dimensional reconstruction of bone structures is frequently available, and numerous recent studies tend to take advantage of this tool to assist surgeons in planning the surgery.

Proximal humerus fractures require “relative” urgent surgical management: the surgeon can rely on the patient’s data, clinical examination, plain radiographs and CT scan images to assess the damages, adopt the most adequate therapeutic strategy and predict the functional outcome after surgery. However long-term prognosis of functional result is challenging, as complications are frequent and jeopardize the results. Boileau *et al.* [3] reviewed proximal humeral fractures treated by hemiarthroplasty with retrospective plain radiographs and CT scan in order to evaluate risk factors for tuberosity complication and poor functional outcome. He identified radiological criteria that can be used to predict good functional outcome: anatomical positioning of greater tuberosity, healing of greater tuberosity around the prosthesis and restoration of the scapulo-humeral arch.

Even though appropriate imaging is mandatory before surgery to establish the appropriate choice of the therapy and estimate the surgical difficulties. Gregory *et al.* [4] advocate the systematic use of pre-operative computed tomography in 3 and 4 part proximal humerus fracture, to analyse fragment displacement and comminution, classify the fracture, assess humeral head vitality, evaluate the mechanical properties of the underlying bone and plan the height of the prosthesis.

### 2.2 Elective Surgery

Total shoulder arthroplasty is the main treatment for advanced shoulder osteoarthritis. Depending on the integrity of the rotator cuff, the surgeon can choose between anatomical and reverse shoulder arthroplasty. The surgical procedure is challenging and its success is linked with a thorough preoperative planning. Beyond the analysis of the rotator cuff status (tendinopathy, trophicity and fatty degeneration) from arthrogram CT, Ultrasound scan or MRI, CT scan three-dimensional reconstructions are crucial to determine the 3D deformation of glenoid due to erosion, occurrence and location of osteophytis and subsequently the centre of the native glenoid and to evaluate the residual bone stock of the glenoid [5, 6]. When associated with osteoabsorptiometry, the 3D subchondral bone density distribution of the arthritic glenoid vault can be addressed [7].

The above mentioned data are critical to plan the operative management of bone loss [8].

In reverse arthroplasty, they determine the viable options in the positioning of the glenosphere [9]. In anatomical shoulder arthroplasty, they insure a correct positioning (version, inclination, rotation, offset) of the glenoid implant, knowing that non-aligned implants lead to increase radiographic loosening rates [10, 11]. However Gregory *et al.* [12] compared preoperative and postoperative CT scans of patient undergoing TSA and showed that the glenoid component positioning strongly depends on the preoperative glenoid erosion.

Authors have recently evaluated innovative surgical methods based on pre-operative CT 3D reconstruction: patient specific instrumentation. Levy *et al.* [13] and Walch *et al.* [14] developed this novel surgical method for placing the glenoid component with the use of patient specific templates created by preoperative surgical planning and 3D models: the principle is to virtually place the glenoid implant on preoperative CT exams with the use of a dedicated software. Then, patient-specific guide is created from 3D printing technology to direct the guide pin into the desired orientation and position in the glenoid during the surgical procedure.

## 3 POSTOPERATIVE IMAGING

### 3.1 Immediate Postoperative Imaging

Standard plain X-rays (true anterior-posterior view and lateral Lamy view) are routinely shots after total shoulder arthroplasty procedure. It allows verification of the correct positioning of the implants (matching, orientation), and provides reference images on which, the follow-up of the patient depends: anything new appearing in the follow-up images can be compared with the first one.

Recently, these plain radiographs have been used in numerous studies to find new immediate postoperative criteria leading to longer lifespan of the implant and improving long term general acceptance, generally using scores (Constant, Oxford shoulder). For instance, Lädermann *et al.* [15] showed that reverse shoulder arthroplasty performed using a deltopectoral approach reduced the length of the arm by 0.5cm than using a transdeltoid approach. But when it comes to active anterior elevation, the transdeltoid approach minimally restricted the angular amplitude by 10°. These results are based on the comparison of preoperative plain radiographs with immediate postoperative images. Without entering the details concerning all the results, early plain radiographs allowed searchers to study numerous other postoperative parameters: anatomic restoration of the humeral head using Copeland shoulder resurfacing arthroplasty versus standard approach [16] or subscapularis sparing approach versus standard approach [17], displacement of the centre of rotation induced by the operation using stemmed or resurfacing method [18], mean neck shaft angle for 3 or 4 part proximal humeral fracture [19] or using novel reverse shoulder arthroplasty [20], and the involvement of scapular neck length in scapular notching after a reverse shoulder arthroplasty [21]. Other imaging techniques have also been used to visualize anatomical elements not seen on plane X-rays. For instance, Felix *et al.* [22] used magnetic resonance imaging and ultrasound systems to assess the wholeness of the subscapularis tendon after an assumed sparing novel technique.

### 3.2 Long-Term Follow-Up

During the subsequent years after surgery, the patient is monitored by evaluation on regular basis intervals. The monitoring includes clinical level of functionality, mostly assessed by scores, angles of shoulder amplitude and radiographic images. The most common complications are: infection, stiffness, remaining pain, shoulder instability, rotator cuff secondary tear and aseptic loosening of the implant. Glenoid loosening continues to be the primary reason for the failure of total shoulder arthroplasty (TSA) [23]. In a metanalysis involving 33 studies and 2540 TSA from 1996 to 2005 [2], the rate of aseptic loosening was reported to be 39% and 83% of those involving the glenoid component

### 3.3 Loosening Mechanisms

In TSA, aseptic loosening emerges mostly from the glenoid implant. The different mechanisms involved are: the rocking horse effect and impingement between the edge of the glenoid rim and humeral metaphysis, specially in the uncovered area [24]. These two mechanisms generate PE particles that induces the deterioration of polyethylene, leading to the development of a polyethylene granuloma causing the aseptic loosening. Numerous aggravating factors encourage these mechanisms [25]. The most important are the quality of the primary fixation of the glenoid implant and its positioning, positioning of the humeral head, mismatch between these two implants, quality of the underlying subchondral bone, roughness of the implants and the cementing technique. Specifically, positioning of the glenoid implant is a critical step for clinical outcomes and long-term lifetime of the anatomic total shoulder arthroplasties [28].

Therefore, preoperative assistance for the implant positioning seems necessary when setting an anatomic total shoulder arthroscopy. One can select the instrument set positioned on the non-damaged areas of the scapula, individual instrument set based on preoperative images (CT scan or MRI), Rapid Prototype instrumentation or navigation systems.

### 3.4 Radiolucent Lines Observed on Plain Radiographs are Not a Reliable Evidence of Loosening

The mean rate of radiolucent lines in series with more than 10 years of follow-up is reported to be 80% [26]. However, the reported occurrence of radiolucent lines varies greatly between the published series (from 0 to 100%) and has proven to be inconsistent.

It is widely admitted that only progressive radiolucent lines are associated with loosening of the glenoid implant. This criteria of “progression” is questionable, since the value of one observation of a radiolucent line is questionable: it is observer dependant, and even a slight change in the incidence of the radiograph can interfere with the RL analyses [26, 27]. Beyond that, X-rays underestimate radiolucent lines. Yian *et al.* [26, 27] studied a series of 47 TSA: 40% of the radiolucent lines visualized with CT scan could not be seen on the plain X-rays. More recently, Gregory *et al.* [26] showed that the inter observer reliability is three times higher from the analysis based on CT scan images rather than the one based on plain X-rays, and 74% of the osteolysis seen on CT scan images could not be seen on the plain X-rays. Thus, the results of the studies based on radiolucent lines from plain X-rays are questionable.

### 3.5 Radiolucent Lines and Osteolysis Seen From CT Scan Images are Linked to the Loosening of the Glenoid Implant

RL analysis based on CT scan images is more reproducible than based on X-rays [27, 28] and allows the periprothetic osteolysis analysis. This osteolysis can be defined by an area free from bone framework wider than 2mm. Gregory *et al.* proposed a five stage score to classify osteolysis around the glenoid implant [28]: absence of osteolysis, osteolysis located to one or two aspect of the fixation, massive osteolysis surrounding the whole fixation with respect to the cortical bone, massive osteolysis with one or more cortical permeation, and massive osteolysis associated to the lysis of the cortical bone. In a sample of 68 TSA followed-up and assessed with CT scan within a 6 to 88 months period (mean 35, SD 26), Gregory *et al.* [28] showed an increase in the radiolucent lines, assessed with both the Molé score, and osteolysis. Clinical results are consistent with deterioration of the fixation of the glenoid implant with time.

Besides that, the connection between radiolucent line assessed from CT scan images and the aseptic loosening of the implant has been confirmed during *in-vitro* studies [29]. In this study, the constraints applied by the humeral head to the coil were repeated on 6 prosthetic coils implanted on cadaveric bone. The loosening evolution was evaluated by iterative CT scans. Later, the implants were cut, and CT scan images were compared to the analysis of the fragment with optic microscope. This comparison showed that the radiolucent lines matched with a loosening of the implant, undergoing eccentric mechanical stress (distraction and compression constraints), showing that the loosening progressed from the periphery of the implant to the centre of the fixation. The involved interface develops first between the implant and the cement, and then lately between the cement and the bone. This last interface leads to the complete loosening of the implant [30].

If the assessment of a radiolucent line on plain radiographs is not strongly conclusive [26], and its evolution is hardly predictable, the revealing of radiolucent lines from CT scan images is associated with the loosening of the implant, partially if it is only restrained to a limited area of the implant, and it is completed when the radiolucent line surrounds the implant [29].

### 3.6 Radiolucent Line, Osteolysis and Clinical Relevance

Even if these radiolucent lines and radiologic osteolysis match with the loosening of the implant, they however, not always lead to functional and clinical loss of shoulder feature. According to Torchia *et al.* [31], the assessment of osteolysis or a complete radiolucent line surrounding the implant and wider than 1.5mm, leads to clinical pain felt by the patient. In many other studies, the clinical restriction of movement was limited compared to the strong radiological loosening signs [32, 33]. This mismatch between the results of the different studies is due to the fact that the study of loosening is based on the analysis of plain graphs, rather than CT scan. As previously noticed, the radiolucent lines observed on plain graphs are not reliable for the assessment of loosening [26, 27]. In 2006, Zilber *et al.* [34] introduced the concept of “floating glenoid” after studying the long term results (15 to 21 years) of a TSA sample; it designates a glenoid surrounded by osteolysis without any functional limitation.

According to Gregory *et al.* [28], the functional limitation (excluding shoulder rotator cuff injuries and/or trauma induced loosening) might be due to the expansion of the osteolysis to the cortical bone with its lysis, inducing destabilisation of the implant, thus provoking pain.

### 3.7 Polyethylene Deterioration, Pace of Deterioration, Polyethylene Granuloma

A CT scan study of 68 TSA [28] showed osteolysis in nearly all the subjects with a follow up over 40 months (24 subjects within 27). There is, to date, no consensus on the significance of these images. Wirth *et al.* [25] performed a histological analysis of the membrane surrounding three TSA retrieved because of aseptic loosening (with major osteolysis on the follow-up X-rays). They found in each subject the same polyethylene granuloma liable for the aseptic loosening of total hip arthroplasties. The difference lied in the shape of the particles (less spherical, more fibrillar). Other authors performed PET CT to assess the biological activity of these images of osteolysis. They found an intense reaction around the implant, where the osteolysis could be seen with the CT scan. It can possibly match with the polyethylene granuloma inflammatory reaction [35].

In another study, the deterioration pace of the polyethylene was studied using CT scan *in vivo* method [36]. Neer 2 (Smith and nephew) implants were assessed. The rate of deterioration of the polyethylene was estimated to be 0.38mm per year (for a 4mm thick implant). Even though the shoulder is not a weight bearing joint, this rate of deterioration is close to those found for total hip arthroplasties (0.1 to 0.4mm per year [37, 38]). Knowing the limited PE thickness of glenoid implants (4 to 5mm), these results might explain why the lifetime of these implants rarely exceeds 10 years. The mechanisms responsible for polyethylene deterioration are therefore especially relevant.

## CONCLUSION

The results of this review suggest that even though imaging is already strongly considered in preoperative and postoperative usage, many applications are yet to be developed and spread. Research efforts are to be made for its promising use concerning highly patient specific materials and techniques based on preoperative CT scan. Moreover, we recommend employing CT scan for the long-term follow-up, specifically to monitor aseptic loosening as it has proven to be more reliable than plain graphs alone.
